# A New Concept for Quantifying the Complicated Kinematics of the Cervical Spine and Its Application in Evaluating the Impairment of Clients with Mechanical Neck Disorders

**DOI:** 10.3390/s121217463

**Published:** 2012-12-17

**Authors:** Chia-Chi Yang, Fong-Chin Su, Lan-Yuen Guo

**Affiliations:** 1 Department of Biomedical Engineering, College of Engineering, National Cheng Kung University, Tainan 701, Taiwan; E-Mails: alexrain0226@hotmail.com (C.-C.Y.); fcsu@mail.ncku.edu.tw (F.-C.S.); 2 Medical Device Innovation Center, National Cheng Kung University, Tainan 701, Taiwan; 3 Department of Sports Medicine, College of Medicine, Kaohsiung Medical University, Kaohsiung 807, Taiwan

**Keywords:** mechanical neck disorder, kinematics of cervical spine, cervical workspace, motion of cervical circumduction, electromagnetic tracking system

## Abstract

Mechanical neck disorder (MND) is one of the most common health issues and is characterized by restricted cervical mobility. However, traditional kinematic information often focuses on primary movement in the cardinal plane, which seems insufficient to fully determine the kinematics of the cervical spine because of the complexity of the anatomical structures involved. Therefore, the current investigation aimed to modify the concept of the three-dimensional workspace to propose an objective mathematical model to quantify the complicated kinematics of the cervical spine. In addition, the observation evaluated the characteristics of the cervical workspace in asymptomatic and MND groups. Seventeen healthy volunteers and twenty-five individuals with MND participated in the study and executed the motion of circumduction to establish the cervical workspace using an electromagnetic tracking system. The results produced a mathematical model to successfully quantify the cervical workspace. Moreover, MND groups demonstrated significant reduction in the normalization of the cervical workspace with respect to the length of the head-cervical complex. Accordingly, the current study provided a new concept for understanding the complicated kinematics of the cervical spine. The cervical workspace could be a useful index to evaluate the extent of impairment of the cervical spine and monitor the efficacy of rehabilitation programs for patients with MND.

## 1. Introduction

Mechanical neck disorder (MND) is one of the most common health issues of the modern lifestyle. It is characterized by the symptoms of neck pain, restricted cervical range of motion (ROM), altered neuromuscular control, neck muscle weakness and neck-related functional disabilities [1–10]. A number of epidemiological studies have provided evidence the prevalence of neck pain in the general population is high, and neck pain could be a serious condition. In Western countries, approximately 30% to 50% of the population has experienced neck pain [11,12]. In the United Kingdom, it was estimated nearly one fifth of adults had suffered from a new episode of neck pain within the previous year [13]. Côté *et al.* found 66% of respondents had experienced neck pain during their lifetime and 54% of respondents had experienced neck pain at some point in the six months prior to the survey [14]. In the Taiwanese working population, a one-year prevalence rate of 24.5% self-reported neck pain was documented [15]. Most importantly, the epidemiological report stated only 6.3% of patients with MND in the previous one year were free of recurrence [16].

In general, the cervical spine is a highly flexible section of the spine. Owing to the complexity of the anatomical structures of the cervical spine, the movements of the cervical spine are three-dimensional and critical to the execution of many daily living activities. In the clinical setting, knowledge of the kinematics of the cervical spine is essential to examine the extent of impairment and evaluate the efficacy of rehabilitation programs for patients with MND. One way to comprehend the kinematics of the cervical spine fully is to quantitatively measure the cervical ROM. According to the recommendation of “Guides to the Evaluation of Permanent Impairment” [17], cervical movement has been commonly subdivided into six primary movements in three major anatomical planes, including flexion, extension, left and right lateral flexion, and left and right rotation. Previous efforts attempted to distinguish differences in the cervical ROM between asymptomatic persons and those with MND, concluding that aberrant cervical mobility is a general phenomenon in patients with MND [2,3]. However, a definitive conclusion regarding which direction is the most affected in patients with MND has still been controversial until now.

Inasmuch as there are no definitive conclusions to clearly distinguish differences in the complicated kinematics of the cervical spine between asymptomatic persons and those with MND, an alternative and feasible method is desperately needed to overcome this problem. Further, traditional methods of assessment for cervical spine attempt to quantify individual cervical ROM in three separate major anatomical planes. No previous studies utilize the concept of combining three major anatomical planes to explore the kinematics of the cervical spine. Recently, the workspace defined as the volume of joint movement in the three-dimensional space had been proposed for application to human joint kinematics [18–20]. The previous observations found that the workspace of the trapeziometacarpal joint could be used to replace the traditional angular parameters and provide a quantitative method to evaluate thumb impairment. Thus, the three-dimensional workspace was modified to apply to the complicated kinematics of the cervical spine. Here, the cervical workspace was operationally formed by the joint center of C7 and the motion trajectories of cervical circumduction, comprised of all the cervical movements in the three major anatomical planes.

To improve knowledge of the complicated kinematics of the cervical spine, the major purposes of the present investigation were to modify the concept of the three-dimensional workspace to propose an alternative and quantitative method for depicting the kinematics of the cervical spine and to find a mathematical relationship to quantify the cervical workspace from the length of the head-cervical complex in healthy subjects. Through the proposed mathematical determination, the ideal amount of cervical workspace would be easily predicted from a given known amount of the length of the head-neck complex. Finally, the observation also evaluated the characteristics of cervical circumduction between the symptomatic and mechanical neck disorder groups. The findings give new insight into the properties of motion of cervical circumduction and further provide an index to determine the extent of impairment for patients with MND.

## 2. Methods

### 2.1. Subjects

Forty-two subjects were recruited for this investigation. Seventeen of these subjects were healthy controls and volunteered to establish the normal database of the maximum cervical workspace. None of the subjects had any history of cervical surgery, cervical trauma or cervical pain. The remaining twenty-five subjects were included in the MND group because they had neck pain.

All subjects with MND had been diagnosed by a physician and had sought medical treatment within the past six weeks. If they showed any circulatory or neurological disorders the subjects were excluded. In addition, participants were also excluded if they were diagnosed with spinal orthopedic problems, such as spinal vertebral fracture, abnormal spinal lordosis, spinal spondylosis or spinal osteoarthritis and so on. All subjects gave informed consent to the experimental procedures, which had been approved by the Human Institutional Ethics Committee prior to participating in the investigations. Additional demographic details were initially recorded for each participant. Thirteen of twenty-five subjects with MND (eight men, five women) completed the Neck Disability Index to evaluate the functional disability [21]. Participants’ characteristics, demographic data presented in Table 1. No group differences were found in gender proportion and other demographic data.

### 2.2. Apparatus

Three-dimensional kinematic data of the motion of cervical circumduction was recorded with a six-degrees-of-freedom electromagnetic tracking system (ETS) (LIBERTY^™^, Polhemus Inc., Colchester, VT, USA). The ETS is a non-invasive measurement tool consisting of a standard range electromagnetic source that generates low-frequency electromagnetic fields detected by one or multiple receiver sensors. The ETS is capable of tracking consecutive positions (X, Y and Z Cartesian coordinates) and the orientations (azimuth, elevation, and roll) of the receiver sensors relative to the electromagnetic source. As a result, the ETS can not only provide dynamic and continuous information, but also simultaneously measure the three-dimensional joint ROM in three planes over the time period of the movement. According to manufacturer specifications, the static root mean square (RMS) accuracy is 0.0762 cm for X, Y or Z position and 0.15° for sensor orientation and the useful operation is in excess of 180 cm (LIBERTY^™^ User Manual, Revision F, 2008). The size of the electromagnetic source size is 5.6 cm × 5.6 cm × 5.8 cm (length × height × width) and the dimension of the receiver sensor is 2.26 cm × 1.27 cm× 1.14 cm (length × width × height). Our previous investigations also demonstrated ETS is appropriate and applicable in quantifying the three-dimensional measurement of cervical kinematics [22].

**Figure 1.**
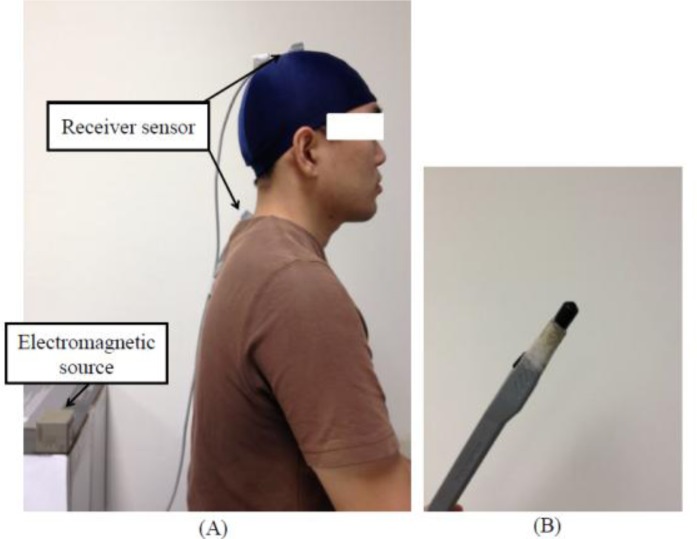
Experimental set-up to collect the movement trajectories of the motion of cervical circumduction: (**A**) Placement of applied electromagnetic source and receiver sensors; (**B**) Another receiver sensor was mounted on a palpation stylus.

In this experiment, the three-dimensional positions of the receiver sensors relative to the electromagnetic source were tracked at a measurement frequency of 120 Hz. To obtain the movement trajectories of the motion of cervical circumduction, three electromagnetic receiver sensors were applied in the experiment; one of these two receiver sensors was placed on the vertex of the head, which was defined as the conjunction point between the bi-auricular and the medial sagittal line by means of an adjustable, plastic hat and the other was firmly attached over the processus spinosus of the seventh cervical spine body (C7). Moreover, another receiver sensor mounted on a palpation stylus, pen-shaped device was used to palpate bone landmarks to define the position of the joint center of C7. The electromagnetic source was positioned near the subject (Figure 1).

### 2.3. Experimental Procedure

All measurements were performed by the same tester in a quiet room. At the beginning of each measurement, each subject was requested to sit straight on a wooden chair to keep his or her thoracic spine in contact with the backrest, relax the cervical spine and look straight ahead. The subject’s feet were flat on the floor, and their arms rested freely on their thighs. First, the position of the incisura jungularis was recorded utilizing palpation stylus and the position of the joint center of C7 was subsequently determined. Next, the subject was instructed to flex the cervical spine and this flexed position was defined as the starting position of the motion of cervical circumduction. Each subject was then asked to execute the actively maximum motion of cervical circumduction as far as possible at a normal velocity, and then return the cervical spine to the starting point.

**Figure 2.**
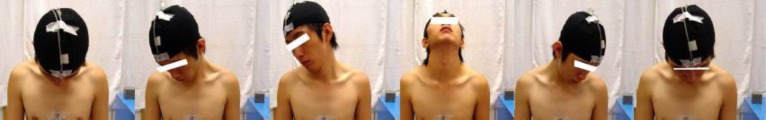
Measurement method for measuring the motion of cervical circumduction: the motion of cervical circumduction was a consecutive combination of flexion, extension, lateral flexion, and slight rotation.

The motion of cervical circumduction was defined as the movement pattern which was a consecutive combination of flexion, extension, lateral flexion, and slight rotation (Figure 2). It meant the head was moved in a smooth continuous arc from the starting position to lateral flexion, followed by extension. Then, the head was executed to contralateral lateral flexion and back to the starting position. The trajectory of the motion of cervical circumduction formed an elliptic-like curve from the transverse plane. The direction of the motion of cervical circumduction was subjectively determined by each volunteer. Each movement was performed consecutively without stopping. No feedback was provided to correct the volunteer’s patterns of movements. Data from three successful trials of each subject was used for analysis and each trial of movements was collected at least five minutes apart.

### 2.4. Data Processing

**Figure 3.**
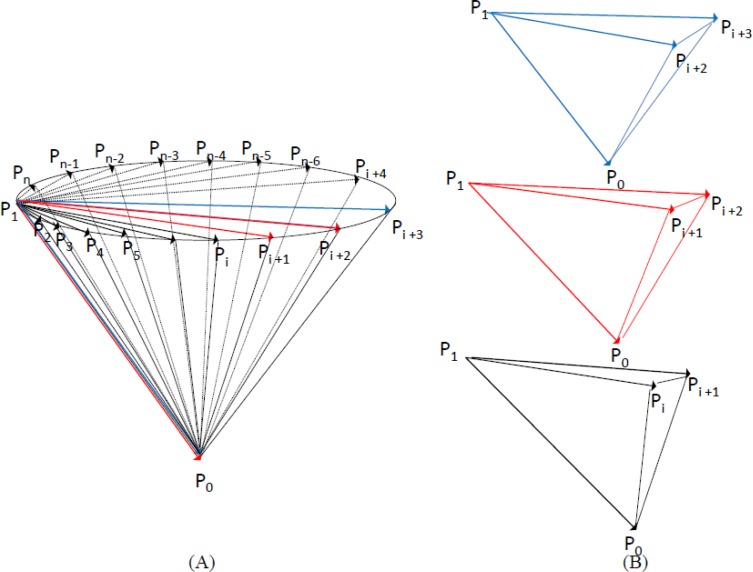
Illustration of the calculation of the volume of the cervical workspace: (**A**) The cervical workspace was modeled by an inverted irregular elliptic cone and composed of a different magnitude of inverted tetrahedron; (**B**) the volume of the cervical workspace was obtained by summing the volume of the individual inverted tetrahedron.

While performing the motion of cervical circumduction, the consecutive position *versus* time data of the attached receiver sensors was collected. Self-developed MATLAB code (Version 7.5.0, The Mathworks Inc., Nattick, MA, USA) was written for processing the data. First, the joint center of C7 was operationally defined as the midpoint between the position of the receiver sensor on C7 and the position of the incisura jungularis based on anatomical structure of the human body [23]. The transformation between the position of the joint center of C7 and the position of C7 could also be determined. The position of joint center of C7 could be further determined through the transformation while executing the motion of cervical circumduction and was regarded as the vertex. Then, the consecutive position data of the vertex of the head relative to the joint center of C7 was used to reconstruct the elliptic base. Hence, an inverted irregular elliptic cone was formed by the position of the joint center of C7 and the consecutive position data of the vertex of the head relative to the joint center of C7 and operationally defined as the cervical workspace (Figure 3). Next, the mathematical determination was proposed to obtain the volume of cervical workspace. Theoretically, since the cervical workspace was composed of a different magnitude of inverted tetrahedron, the volume of the cervical workspace was obtained by summing the volume of the individual inverted tetrahedron. The cervical workspace was computed according to the following equation:

$$\text{Cervical}\;\text{workspace}=\sum_{i=1}^{\text{n}-2}\left\{\frac{\left|\left(\vec{{\text{P}}_{0}}-\vec{{\text{P}}_{1}}\right)\cdot \left[\left(\vec{{\text{P}}_{\iota +1}}-\vec{{\text{P}}_{1}}\right)\times \left(\vec{{\text{P}}_{\iota +2}}-\vec{{\text{P}}_{1}}\right)\right]\right|}{6}\right\}$$

where n was the number of position data of the vertex of the head collected in this experimental trial; P_0_ was the position of the joint center of C7; P_1_ was the starting position of the vertex of the head and P_i_ denoted the position data of the top of the head at the *i*th sample. On the other hand, to clarify whether a proportional relationship exists between the cervical workspace and the length of the head-cervical complex, the distance between the joint center of C7 and the vertex of the head was utilized to represent the length of the head-cervical complex. Meanwhile, to eliminate the effects of the length of the head-cervical complex, the normalization of the cervical workspace with respect to the length of the head-cervical complex was also used for further analysis.

### 2.5. Data Analysis

Data were analyzed using the Statistical Package for Social Sciences (SPSS 12.0, Chicago, IL, USA). Descriptive statistics (mean ± standard deviation) were calculated for demographic data and the measurements of the cervical workspace and the normalization of the cervical workspace with respect to the length of the head-cervical complex for each group. To clarify the effect of the length of the head-cervical complex upon the normal cervical workspace for healthy subjects, a simple linear regression analysis was applied to produce a mathematical equation describing the relationship between these two factors. Since the data from the current study was not normally distributed according to the Shapiro-Wilk test (*p* > 0.05), nonparametric Mann-Whitney *U* test was used to identify the differences in the normalization of the cervical workspace with respect to the length of the head-cervical complex between the symptomatic and control groups. A significance level of *p* < 0.05 was used in all analyses.

## 3. Results

**Figure 4.**
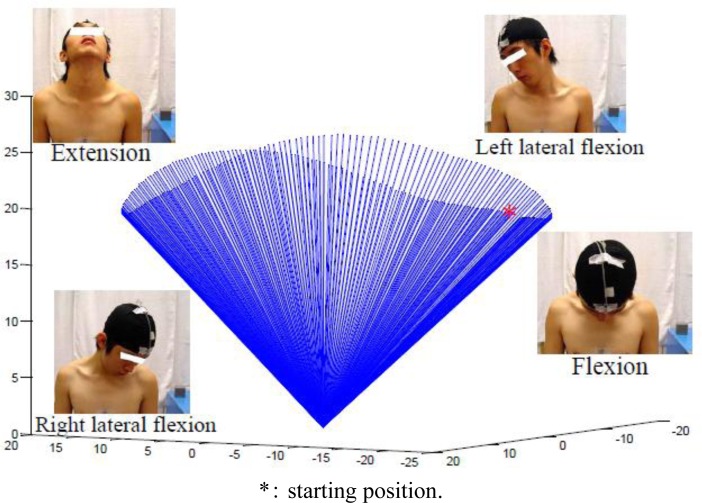
Graphic display of the cervical workspace for one subject (unit: cm).

The contour of motion of cervical circumduction was successfully modeled by the alternative method proposed in the current study. In the proposed graphic display of the cervical workspace, the major motion including flexion, extension, right and left lateral flexion could be easily illustrated in Figure 4.

**Figure 5.**
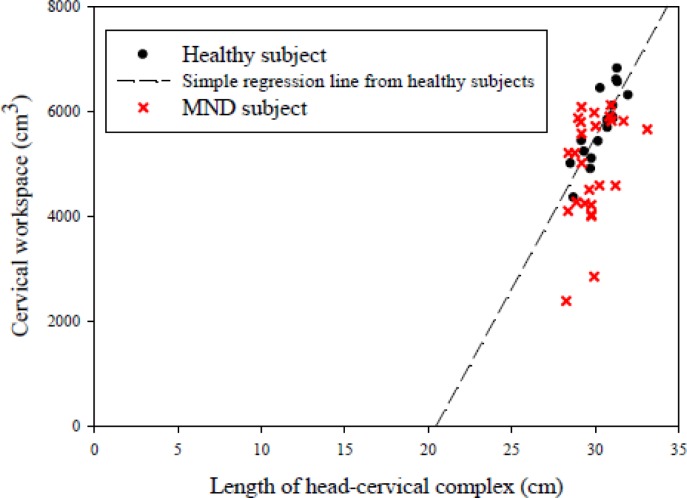
Scatter plot of the cervical workspace *versus* the length of the head-cervical complex for healthy and MND subjects. The simple linear regression equation obtained from healthy subjects: Cervical workspace (cm^3^) = 577.13 × (Length of the head-cervical complex) – 11,814.82 (cm) (*R*^2^ = 0.701, *P* < 0.01).

The mathematical determination demonstrated the mean value (±standard deviation (SD)) of the cervical workspace for healthy subjects was 5,726.33 (688.87) cm^3^. Simple linear regression analysis showed the cervical workspace was significantly correlated with the length of the head-cervical complex (*R^2^* = 0.701, *P* < 0.01) for healthy subjects. In other words, a proportional relationship was found between the cervical workspace and the length of the head-cervical complex and could provide a quantitative method of calculating the ideal amount of cervical workspace given a known length of the head-cervical complex. The equation derived from a simple linear regression analysis was as follows:

$$\text{Cervical}\;\text{Workspace}\;({\text{cm}}^{3})=577.13\times (\text{Length}\;\text{of}\;\text{the}\;\text{head}-\text{cervical}\;\text{complex})-11814.82(\text{cm})$$

where *R^2^* was 0.701. A scatter plot of the cervical workspace *versus* the length of the head-cervical complex for healthy and MND subjects and the fitted regression line obtained from healthy subjects is shown in Figure 5.

In addition, because the results from linear regression analysis distinctly suggested the length of the head-cervical complex could influence the cervical workspace, the normalization of the cervical workspace with respect to the length of the head-cervical complex was used to further analysis. Expectedly, a significant decrease in the normalization of the cervical workspace (*P* = 0.037) was identified in the MND group (Table 2).

## 4. Discussion

In a clinical setting, the kinematics of the cervical spine are useful and essential for basic understanding of cervical biomechanics and the mechanisms of cervical dysfunction. Unfortunately, traditional angular parameters seem to be unable clarify the complicated kinematics of the cervical spine clearly and thoroughly due to the complexity of anatomical structures of the cervical spine. Therefore, the major aims of this investigation were to modify the concept of the three-dimensional workspace to find an alternative and quantitative method to describe the complicated kinematics of the cervical spine and attempt to identify the proportional relationship between the cervical workspace and the length of the head-cervical complex for healthy subjects. Next, the characteristics of cervical circumduction were used to compare between the asymptomatic and mechanical neck disorder groups. As expected, a mathematical model was obtained to successfully quantify the cervical workspace. The mathematical determination to assess the cervical workspace provided a feasible and alternative manner for characterizing the complicated kinematics of the cervical spine, and what's more, the results of this study supported our hypothesis that a proportional relationship existed between the cervical workspace and the length of the head-cervical complex for healthy subjects. It was tentatively concluded the simple linear regression equation obtained from our experimental data was able to predict the ideal amount of cervical workspace for a given known length of the head-cervical complex for healthy subjects. Another important issue was to characterize the properties of the cervical workspace between the asymptomatic and MND groups. Undoubtedly, the findings of this current investigation demonstrated the normalization of the cervical workspace with respect to the length of the head-cervical complex was significantly reduced in the MND group compared to the asymptomatic group.

Since mechanical neck pain is one of the common health issues in the general population, many previous studies have attempted to clarify the different aspects of the biomechanical properties of the cervical spine in the neck pain population [1–7]. One well-known feature related to neck pain is the trend of restricted cervical ROM in patients with MND compared to healthy subjects [1–3,6–10]. On the other hand, cervical motion is mainly composed of three major anatomical planes of movements, which are commonly subdivided into the six primary movements as follows: flexion, extension, left and right lateral flexion, and left and right rotation because of the complexity of the anatomical structures of the cervical spine. A review of previous studies revealed that inconsistent findings. The findings of Jordan *et al.* and Chiu *et al.* showed only a significant difference in extension between patients with neck pain and healthy volunteers [6,9]. Lee *et al.* and Johnston *et al.* indicated the decreased range in rotation could be observed in patients with neck pain [1,8]. Our previous investigation demonstrated subjects with MND had significantly decreased ROM in right rotation and extension compared to the healthy group [2]. Similarly, it was also found flexion is the affected direction. Hagen *et al.* pointed out flexion and left axial rotation differed significantly between men who reported pain in the past seven days and those with no pain [10]. Additionally, Woodhouse *et al.* suggested chronic neck pain patients had significant reduced motion in all major anatomical planes compared to the asymptomatic controls [3]. Based upon the findings stated above, the conclusion regarding which direction is the most affected one in neck pain subjects remains obscure until now. Consequently, it is difficult to fully understand the biomechanical characteristics of the cervical spine in patients with MND. To solve this problem, a simple and convenient method of quantitating the kinematics of the cervical spine was proposed in this study. Unlike traditional methods of assessment for the cervical spine, the motion of cervical circumduction included all the cervical movements of the three major anatomical planes in a three-dimensional space. Here, the joint center of C7 was regarded as the vertex. The cervical workspace was modeled by an inverted irregular elliptic cone, which was formed by the joint center of C7 and the path of the motion of cervical circumduction. Simultaneously, the graphic representation could be created to describe the motion pattern of cervical circumduction and calculate the volume of the cervical workspace. The mathematical method was available to characterize the properties of the motion of cervical circumduction. Therefore, the main novel contribution of our study was the cervical workspace, combining the movements of all major anatomical planes in a three-dimensional space, and could offer another feasible way to quantify the complicated kinematics of the cervical spine instead of using the angular measurements of different anatomical planes.

According to the results, there was a proportional relationship between the cervical workspace and the length of the head-cervical complex for healthy subjects that could be established by simple linear regression analysis. The results of statistical analysis showed the coefficient of determination (R-square) of the regression model was 0.701, indicating 70.1% of the variance in the cervical workspace could be accounted for by the known variance in the length of the head-cervical complex. That is to say the ideal amount of cervical workspace could be easily predicted from a given known length of the head-cervical complex for healthy subjects. For instance, the length of the head-cervical complex is 30 (cm) for a subject. It therefore could be predicted the ideal cervical workspace would be 5,499.1 (cm^3^). Comparisons between the ideal cervical workspace predicted from the simple linear regression equation and the actual cervical workspace obtained from the clinical setting could provide an important index to evaluate the extent of impairment of the cervical spine or monitor the efficacy of rehabilitation programs for patients with MND.

Recently, there had been increasing evidence suggesting altered motor control strategies in patients with MND [1–3,24–26]. Moreover, our ultimate aim of the current investigation was to distinguish the properties of the motion of cervical circumduction between the MND and asymptomatic group. Unquestionably, the present study exactly showed a significant difference in the normalization of cervical workspace with respect to the length of head-cervical complex was found between MND and asymptomatic group. Since the normalization of the cervical workspace with respect to the length of the head-cervical complex was used to further analysis, the effects of the length of the head-cervical complex on the normalization of the cervical workspace should be eliminated as far as possible. In other words, the length of head-cervical complex should not be the main cause that would lead to difference in the normalization of the cervical workspace. One possible factor contributing to the difference could be the restricted primary and conjunct range of motion in patients with MND [1–3,6,8–10]. Also, the subjects with MND often exhibited altered neuromuscular control strategies of neck muscle [26–29] and this phenomenon would cause reduction in the normalization of the cervical workspace. Another potential reason might be persistent painful condition in patients with MND may contribute to increased disability and restricted cervical mobility aimed at avoiding muscular strain and occurrence of painful movements [30]. Meanwhile, the graphic display of the cervical circumduction was easily used to compare the divergence in motion pattern between the MND and asymptomatic group.

Besides, the current study had certain potential limitations. First, although the current observation demonstrated that the proposed mathematical method would be a feasible and alternative way to quantify the complicated kinematics of the cervical spine and our previous finding suggested the apparatus was appropriate in assessing the three-dimensional mobility of cervical kinematics [22], a test-retest reliability of the proposed method was not conducted in the present work. Further work will focus on the test-retest reliability of the proposed method. Second, volunteers with poor proprioception might have larger repositioning errors while executing the motion of cervical circumduction from the starting position to the ending position due to the difference in proprioception. The phenomenon might cause experimental biases. Finally, the current investigation was based on small and particular samples. The results of the present study might not exactly reflect the cervical workspace for the general population. As a result, the next step will be to increase the sample size to confirm our findings.

## 5. Conclusions

A feasible and alternative mathematical model to quantify the complicated kinematics of the cervical spine was obtained. Through this alternative and quantitative method, the ideal amount of cervical workspace could be predicted for a known length of the head-cervical complex. Especially, the cervical workspace combines the cervical movements of all the major anatomical planes in a three-dimensional space. The findings from the present study provided a new concept for understanding the kinematics of the cervical spine. Moreover, the current observation demonstrated differences in the properties of the motion of cervical circumduction between the MND and asymptomatic group. It is reasonable to expect the proposed approach is useful in providing an important index to evaluate the extent of impairment of the cervical spine and monitor the efficacy of rehabilitation programs for patients with MND.

## Figures and Tables

**Table 1.** Demographic data of healthy and MND subjects.

**Characteristics**	**Healthy control (n = 17)**	**MND group (n = 25)**	***P*-value**
Men	9	14	0.845 ^d^
Women	8	11	
Age (years)	23.7 (4.0)	25.7 (4.4)	0.091 ^e^
Height (cm)	166.2 (8.5)	166.5 (8.5)	0.908 ^e^
Weight (kg)	62.4 (11.8)	59.9 (9.2)	0.581 ^e^
BMI^a^	22.5 (3.0)	21.5 (2.3)	0.337 ^e^
NDI^b^		20.3 (5.3)^c^	

a BMI: Body mass index;

b NDI: Neck disability index;

c eight men and five women with MND completed the Neck Disability Index;

d Chi-square test was used to verify gender proportion between healthy and MND groups;

e Mann-Whitney *U* test was used to identify the differences in age, height weight and BMI.

**Table 2.** Comparison of the normalization of the cervical workspace with respect to the length of the head-cervical complex and the length of the head-cervical complex between healthy subjects and patients with MND.

	**Healthy subjects**	**Subjects with MND**	***P*-value**
Normalization of the cervical workspace (cm^2^)	187.99 (17.83)	165.22 (32.79)	0.037 *
Length of the head-cervical complex (cm)	30.39 (1.01)	29.83 (1.14)	0.056

* A significant level at 0.05.
